# History of the early development of aromatase inhibitors for treatment of breast cancer

**DOI:** 10.1007/s10549-026-08059-4

**Published:** 2026-08-20

**Authors:** R. J. Santen, L. M. Demers

**Affiliations:** 1 Division of Endocrinology and Metabolism, Department of Medicine, University of Virginia School of Medicine, Charlottesville, VA 22908 USA; 2 Department of Pathology, Penn State University School of Medicine, Hershey, PA 17033 USA

**Keywords:** Aromatase, Inhibitors, Breast cancer, History, Estrogens, Estrogen receptor

## Abstract

**Purpose:**

To review the early history of the development of aromatase inhibitors for treatment of breast cancer by the Penn State Hershey collaborative group and to acknowledge the support and advice of Dr. William L. McGuire in the studies conducted.

**Methods:**

The historical information reported here reflect the memories of Drs. Richard J. Santen and Laurence L. Demers and comprehensive documentation provided in the literature references cited.

**Results:**

The project of development of aromatase inhibitors began with the mis-assumption that the regimen of aminoglutethimide and hydrocortisone represented a “medical adrenalectomy” and primarily represented the blockade of adrenal steroidogenesis. Later studies demonstrated that aminoglutethimide blocked the aromatase enzyme by 95–98%. A key research step involved isotopic kinetic studies in post-menopausal women using ^3^H-androstenedione and ^14^C estrone to precisely measure aromatase under equilibrium conditions. Dr. William L. McGuire provided major advice to the investigators, suggested enlistment of key collaborators, provided laboratory assistance in measuring ER levels, and facilitated funding for the studies. Observational studies involved 147 patients treated with the AG/HC regimen and the results demonstrated efficacy of the regimen. Later, randomized controlled trials demonstrated the equivalence of this regimen to surgical adrenalectomy and tamoxifen. In order eliminate the need for HC, three selective inhibitors of aromatase were developed and shown to induce remissions statistically significantly greater than achieved with the anti-estrogen, tamoxifen.

**Conclusion:**

These studies provided definitive evidence of the superiority of aromatase inhibitors over tamoxifen in postmenopausal women with hormone responsive breast cancer.

**Supplementary Information:**

The online version contains supplementary material available at https://doi.org/10.1007/s10549-026-08059-4.

## Introduction

Dr William L. McGuire devoted his career to developing an in-depth understanding of breast cancer [1]. He recognized that a greater understanding of the pathophysiology of breast cancer represented a major challenge but nonetheless was amenable to the use of emerging molecular, imaging, and biochemical techniques. He understood that only with a better understanding of this cancer could novel treatments be developed. He had the vision to recognize that a focused journal dedicated to this purpose would be a mechanism to encourage breast cancer research. Accordingly, he established this journal, **Breast Cancer Research and Treatment**. McGuire also recognized that the identification of talented young investigators, nurturing them through mentorship, and helping them with research funding would leverage his ability to contribute to the progress in breast cancer management. As an example of this, McGuire recognized the importance of the early studies on development of aromatase inhibitors for breast cancer by a group of young investigators at the Penn State Hershey Medical Center. Accordingly, he provided encouragement, advice regarding collaborations, laboratory assistance and strong support for their studies. This treatise provides a detailed history of how the Penn State investigators, Drs. Harold Harvey, Allan Lipton, Eugeniusz Samojlik, Steven Santner, Laurence Demers, Richard Santen and several Endocrine Fellows, first developed aromatase inhibitors for treatment of patients with hormone dependent, estrogen receptor positive (ER+) breast cancer.

**Fig. 1:**
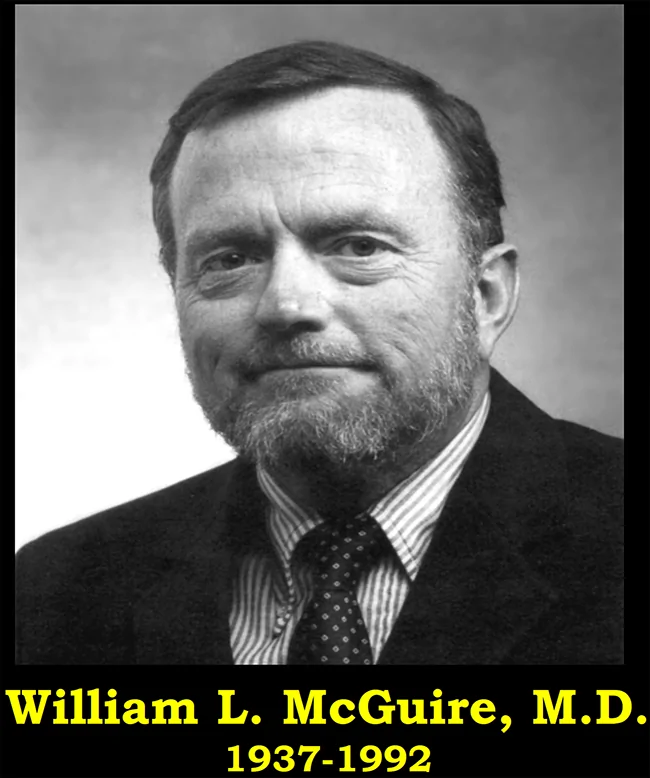
Dr William McGuire was a leader in the field of hormonal treatment of breast cancer. His laboratory ran estrogen receptor assays that were used widely in the United States. McGuire published data demonstrating the importance of the progesterone receptor. He started the journal, Breast Cancer Research and Treatment which has been a leading journal over the last 4 decades

This is a story about research collaboration, synergistic interactions, and substantial federal funding that were key elements in developing a novel therapy for breast cancer. The aromatase development project began soon after the opening of the Penn State Hershey Medical Center Hospital in 1970. Several key faculty members were recruited who served as the collaborative team. The studies over a period of two decades provided a fundamental basis for the large international trials comparing aromatase inhibitors with tamoxifen (Fig. 1).

## History

The story begins when Dr. Richard Santen was an endocrinology fellow at the University of Washington in Seattle, Washington in 1970. While on hospital rounds, he saw a 60-year-old post-menopausal woman with breast cancer who was being treated with high doses of prednisone. This form of “Medical Adrenalectomy” had previously been shown to result in tumor regressions in one-third of patients. The patient appeared very cushingoid: with a buffalo hump, supraclavicular fullness, a chipmunk appearance to her face, multiple bruises, a marked reduction in muscle strength, high blood pressure, and purple striae on her abdomen. She was having depressive symptoms and difficulty sleeping at night. With her breast cancer and all of these physical findings and emotional distress, she was quite miserable. That led Santen to suggest that there might be a better way to block the adrenals and that a drug called aminoglutethimide (AG) might do it. It was known at that time that AG blocked cholesterol side chain cleavage and probably also other enzymes further along in the steroid pathway leading to the production of hydrocortisone by the adrenals. Santen presented this idea to Dr. Alvin Paulson, the Chief of Endocrinology at the Public Health Hospital in Seattle, Washington who immediately responded that this was an impractical idea. However, Santen did not accept this advice and thought that at some time it might be possible to test out this hypothesis.

After finishing his fellowship at the University of Washington, Santen decided to go to the Hershey Medical Center, Penn State University, to work with Dr. Wayne Bardin, a visionary, innovative scientist. Shortly after his arrival, Santen met with Dr. Alan Lipton the head of clinical oncology who had a strong interest in cancer research. During their initial meeting, Lipton asked, “Dick (Santen), do you have any novel ideas about treating breast cancer?” The answer was yes. Together they decided to treat selected breast cancer patients with AG as well as dexamethasone, as glucocorticoid replacement, to block the production of steroids capable of conversion to estrogen. This resulted in a different type of “Medical Adrenalectomy”, one without the use of very high dose prednisone. The assays for estrogen at that time involved radio-immunoassay’s that were not sufficiently sensitive and could not measure very low estrogen levels. Accordingly, the Penn State Hershey Group (PSHG) followed the patient’s clinically to see if their tumors might regress and measured hydrocortisone (HC) levels to monitor the level of adrenal suppression.

Lipton referred one of the first patients, HR, to the Endocrine Clinic for the AG “Medical Adrenalectomy” approach. She was a 65-year-old woman who had severe metastatic breast cancer involving her cervical vertebra and much of her pelvis. She experienced severe pain and weakness necessitating use of a wheel chair. She was found to have complete dissolution of the 7th cervical vertebra and part of her pelvis. The level of stability at the neck was so limited that she was at risk of transecting her spinal cord, leaving her paralyzed. She lived in Ephrata, PA and the roads between the Hershey Medical Center and her small Village were very hilly and quite bumpy. We did our best to try and convince her to undergo the surgery. At the same time, we reviewed with our experimental therapy to block the ability of her adrenal glands to make estrogen precursors. We strongly recommended that she undergo neurosurgery with stabilization of her cervical vertebrae. She was a Mennonite and a very religious woman and told us that she was going to pray to get advice whether to have surgery or use the experimental treatment. She came back to us a few days later and said that God had told her that she should try the experimental treatment first to see if it worked.

**Fig. 2:**
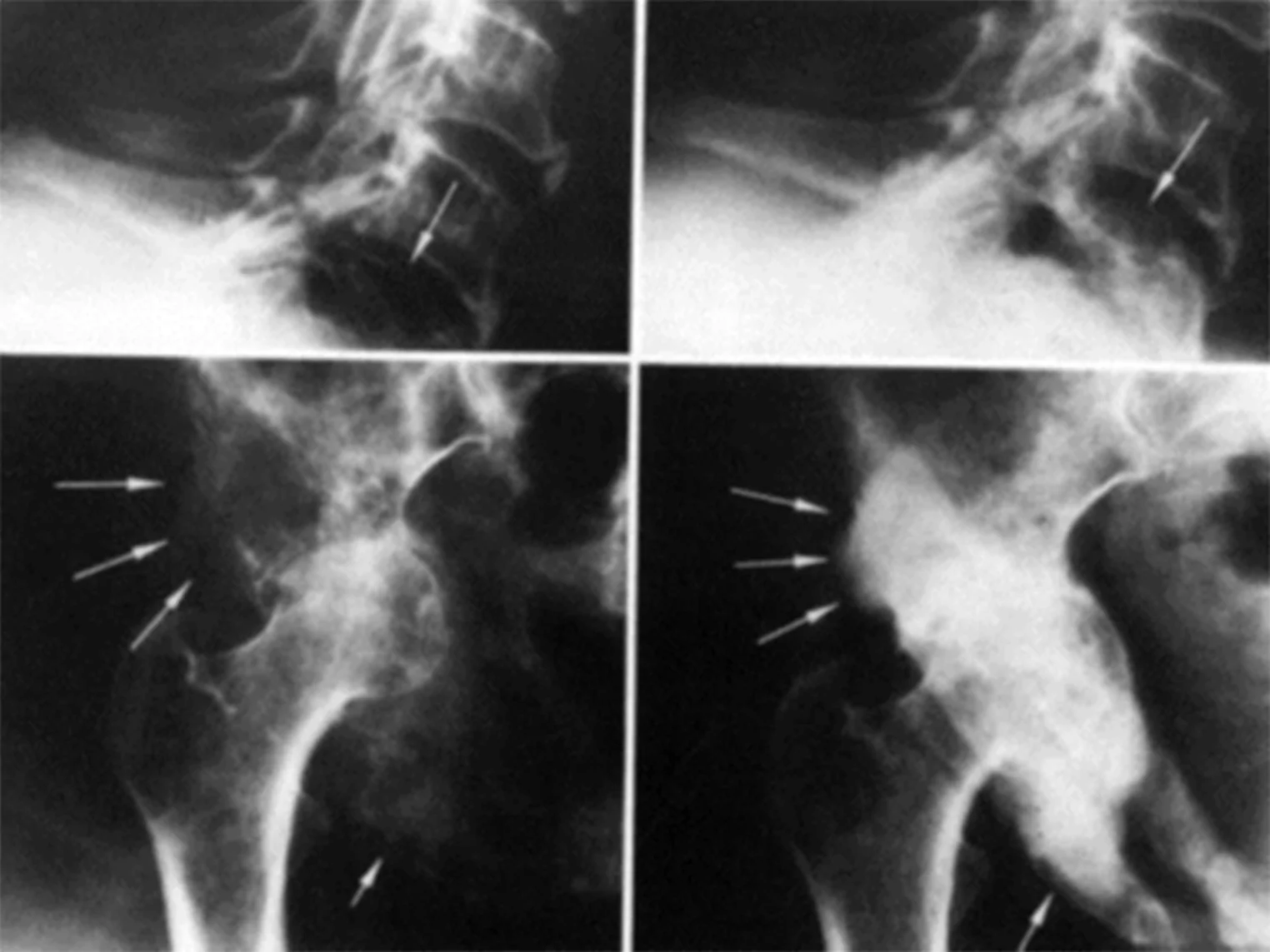
Photos of lytic lesions of the 7th cervical vertebra on the top left panel and hip on the bottom left panel (arrows showing lytic lesions). On the right panels are shown the healing of these areas as reflected by the new white areas representing new bone formation and recalcification. Reprinted from Endocrine Reviews with the permission of the publisher [2]

Very shortly after beginning this experimental therapy, she told us that her pain had gone and that she was feeling much better. About 3 months later she was able to walk without her wheelchair and was pain-free. Another 3 months later we took x-rays of her pelvis and cervical spine and were quite surprised to see that her lytic lesions were completely filled in with calcium suggesting that she had new bone formation there (Fig. 2).

The photograph shown above illustrates the X-rays before and after treatment. The arrows on the left panel show lytic lesions and on the right panel, the healing that occurred after 6 months. This experience convinced us that this therapy worked and likely would work in others.

Soon after, another patient treated with AG further responded dramatically. This patient, a 70-year-old woman, came in with local recurrence of breast cancer on her chest wall (Fig. 3a).

**Fig. 3a:**
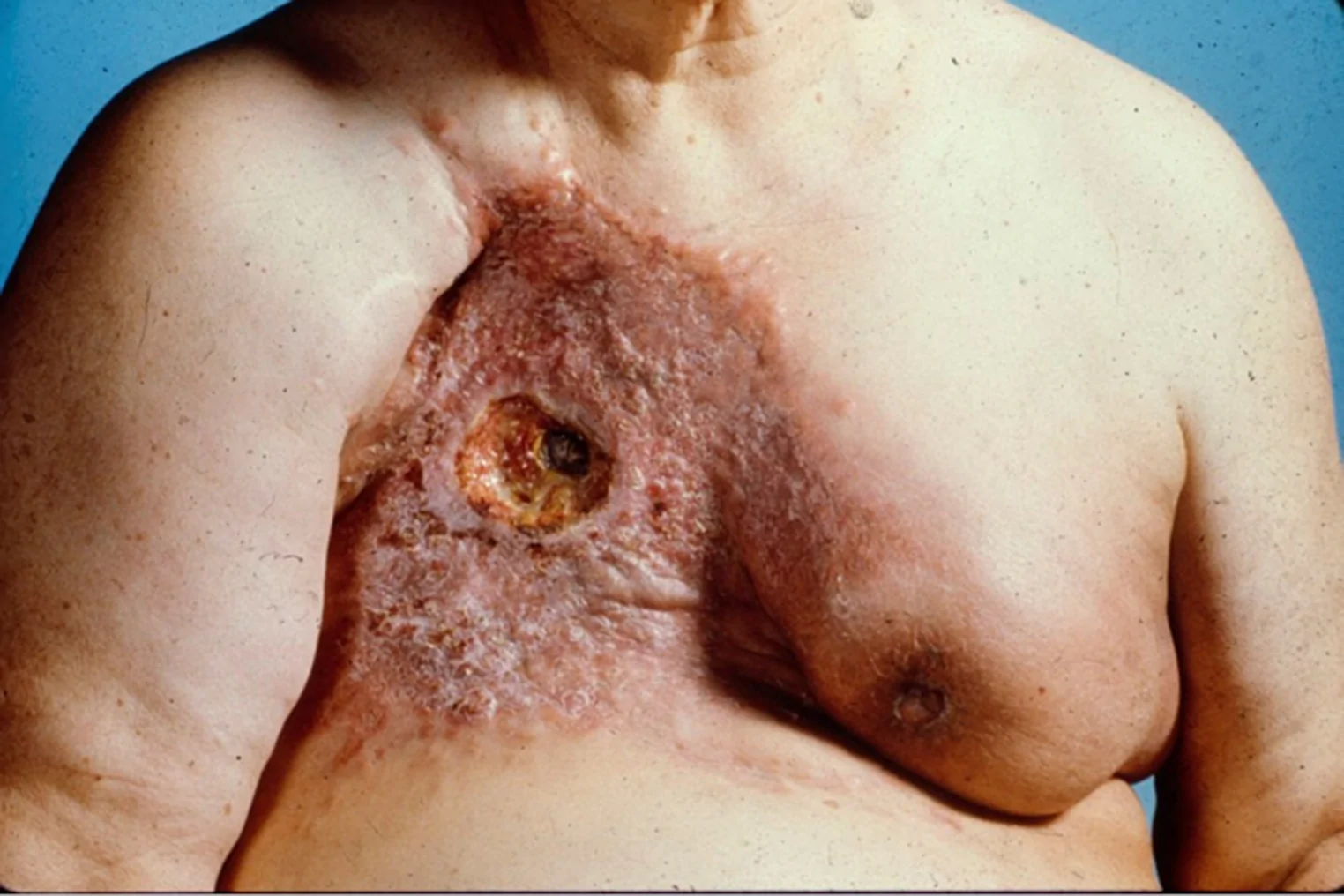
Breast cancer skin lesions before treatment with with aminoglutethimide and dexamethasone

After three months of AG/dexamethasone treatment, these lesions were completely gone. Without a large clinical trial, these two anecdotal cases provided strong evidence of the clinical efficacy of this approach (Fig. 3b).

**Fig. 3b:**
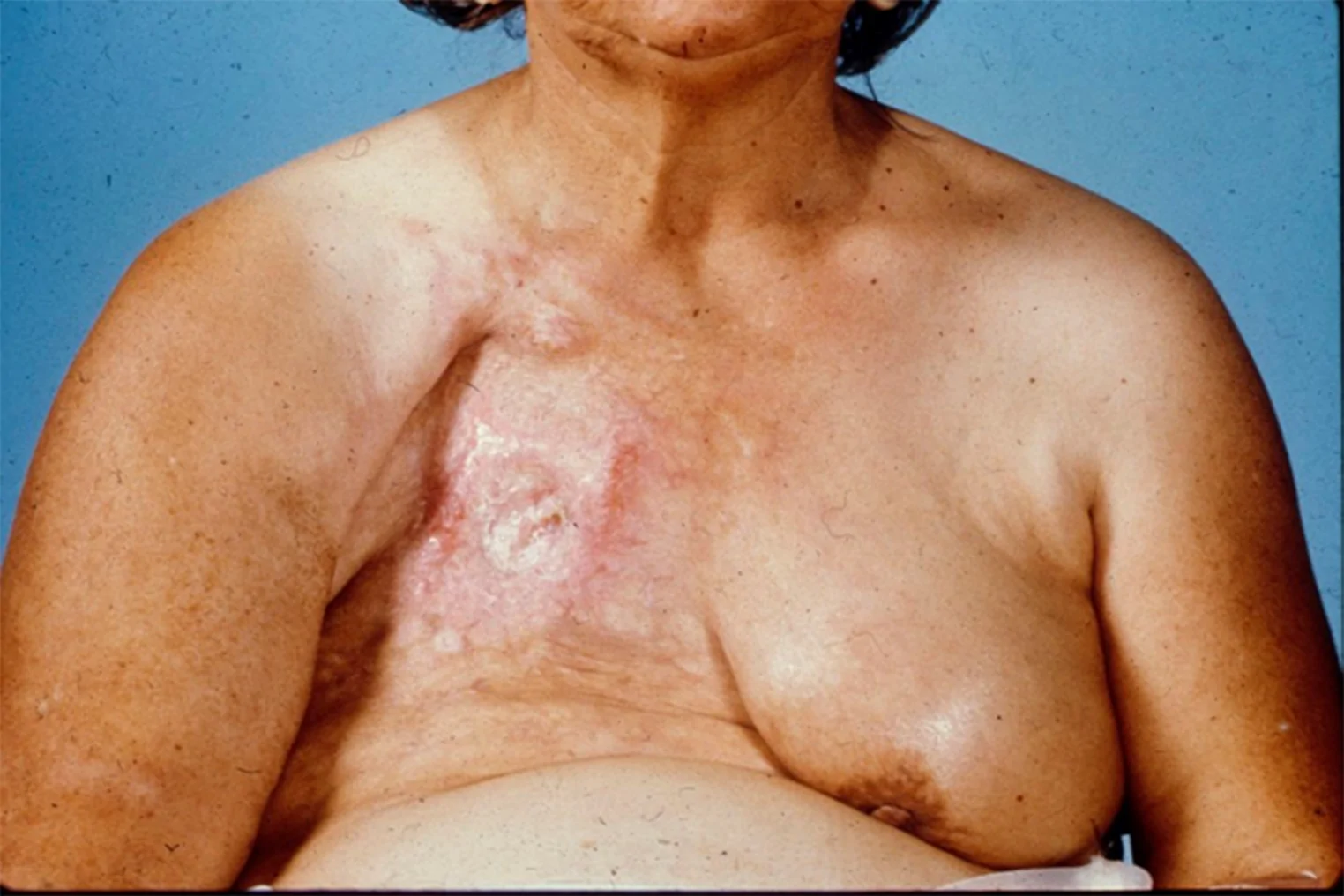
Breast cancer skin lesions during treatment with aminoglutethimide plus dexamethasone

One of the problems with AG was that patients rapidly developed sleepiness. We knew that that a close derivative of AG, called Doriden^R,^ was a sleeping pill. One of the first patients that we treated developed severe lethargy and sleepiness on AG. However, 3 to 4 weeks later the symptoms resolved. That led Demers and Santen to think that the drug might have resulted in increased metabolism of AG. Demers then developed an assay for AG which demonstrated that AG accelerated its own metabolism [3]. This led us to start the AG dose at a very low level and then increase the dose weekly to accommodate to the increased metabolism. Thereafter, sleepiness rarely occurred.

To monitor therapy and prove that the dexamethasone was not the mechanism of responses, we measured plasma cortisol and ACTH levels. From his time in Seattle, Santen knew Dr John Kendall who had developed one of the first radio-immunoassays for ACTH and Kendall agreed to measure ACTH [4]. We found that patients initially had suppression of cortisol but after about 2 weeks the patient escaped from therapy due to elevations in ACTH. This finding led us to determine that AG also increased the rate of metabolism of dexamethasone [4, 5]. We then accounted for this by gradually increasing the dose of dexamethasone, ultimately to 2 mg daily. On this regimen of AG and dexamethasone both the plasma cortisol and the urinary free cortisol levels were suppressed and ACTH levels remained normal. The normal ACTH levels provided evidence that the dexamethasone dosage was not supraphysiologic. We subsequently learned that 6-beta-hydroxylation was up-regulated by AG as the cause of accelerated dexamethasone metabolism. Further, the 6-beta hydroxylation of HC only increased its metabolism minimally, by 15%. Accordingly, we decided to use HC rather than dexamethasone and gave patients 40 mg of HC daily along with the AG [5]. This was higher than the usual replacement dose of HC but the dose was designed to overcome the 15% increased rate of HC metabolism.

**Fig. 4:**
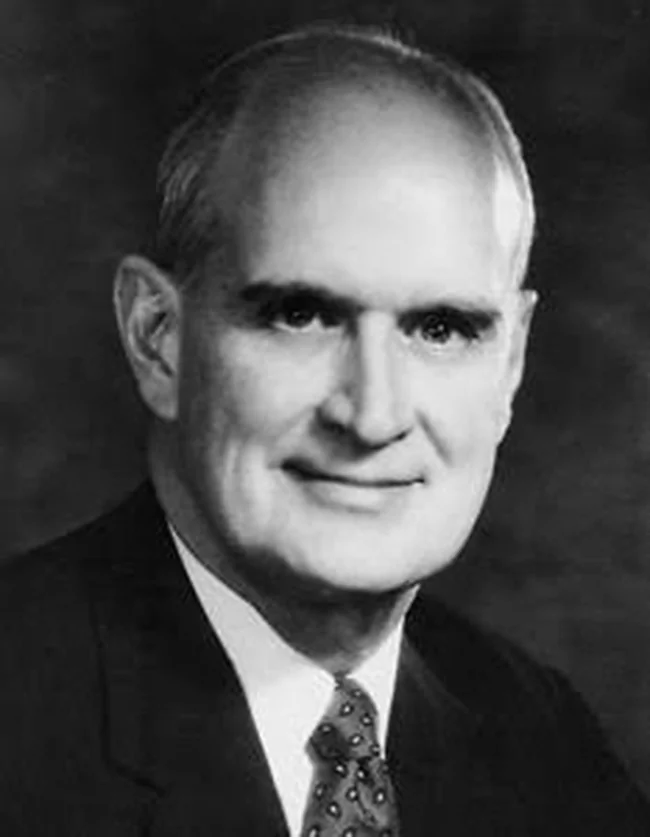
Dr Samuel Wells was a giant in the academic circles of surgery in the United States. He became Chairman of the Surgery Department at Washington University in St Louis and then President of the American College of Surgeons

We treated about 20 patients with the AG/HC regimen and found that about one third responded dramatically with regression of their breast cancer metastases. After presenting these preliminary data in Atlantic City, Dr. William McGuire at Bardin’s suggestion contacted Santen and rendered some important advice. McGuire knew that Dr Samuel Wells at Duke University was conducting clinical trials in patients with breast cancer and suggested that Santen contact him. With the encouragement of McGuire, Dr. Wells decided to collaborate with the PSHG using its AG/HC regimen and entered many patients into treatment at Duke [6] (Fig. 4).

At the time, McGuire chaired the NIH Breast Cancer Task Force and advised the PSHG to apply for a contract to support its work. The PSHG including Harvey, Lipton, Demers, and Santen submitted a contract application to the NIH asking for $250,000 yearly. Based on calculations reflecting inflation, this would represent about $1,688, 742 in 2026. With such a large amount of funding, we could support about ten people to work in the laboratory and to take care of the patients. Sometime after a site visit, we received notice that we were awarded the full $250,000 for the first year with renewal each year based on our progress. As it turned out, we were funded for the next ten years.

**Fig. 5:**
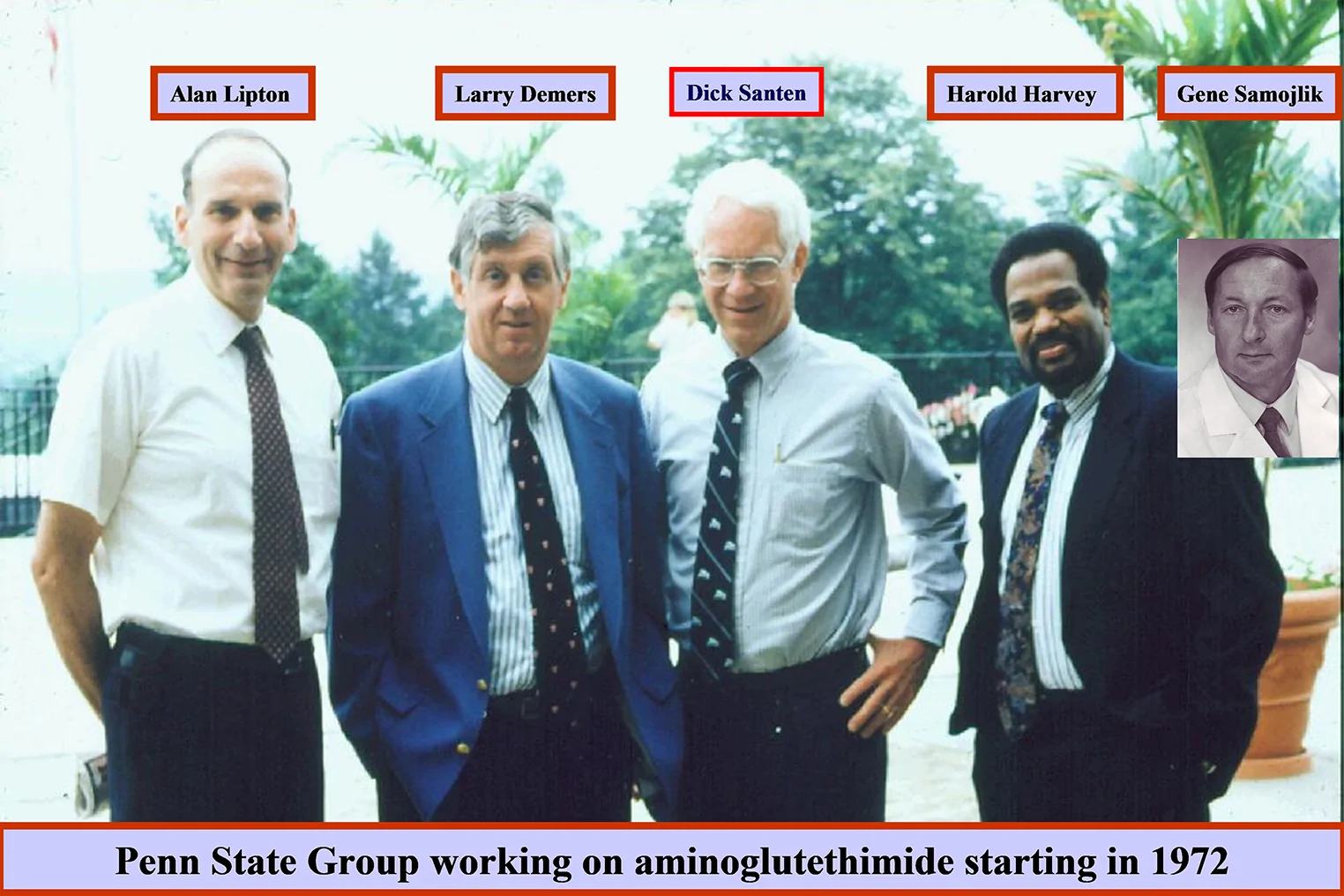
Research is a team effort and the Hershey team worked together to develop aromatase inhibitors for treatment of breast cancer. Left to right are Lipton, Demers, Santen, Harvey and Samojlik

The first order of business was to develop sensitive and specific assays for estrogens. We knew of an individual at the NIH who was known for his expertise in hormone assays, Dr Eugeniuz Samojlik. Gene was recruited to Hershey and over time developed assays to measure both plasma and urine estrone and estradiol. These assays were very labor intensive, involving celite column chromatography. It took Gene several months to establish and validate very specific estrogen assays. He also developed assays for DHEA-S, androstenedione, and several other steroid precursors of HC [7–12] (Fig. 5).

Dr. Samojlik, recognized as an expert in steroid assays, was later recruited to Rutgers Medical Center by Dr. Marvin Kirchner. As the PSHG studies continued to require the ability to measure a large number of diverse hormones, Santen and Demers collaborated to set up a “Core Endocrine laboratory—CEL”. The CEL allowed a comprehensive and concerted approach to measuring all of the key hormones affected by AG. In addition, Demers’s expertise in column chromatography and radioimmunoassay provided novel assays for the evaluation of patients with endocrine related problems in the Medical Center Hospital and Clinics. In addition, the CEL developed an international reputation and performed assays for clinical research and patient care for medical centers throughout the US and Europe [13].

A major advancement in breast cancer research at that time was utilized to enhance our studies. Dr Elwood Jensen from the University of Chicago had developed an assay for the Estrogen Receptor (ER) and had accumulated preliminary data that its measurement could help in the identification of patients whose breast cancers were estrogen dependent. McGuire had the vision to recognize that this assay would provide a major tool in the management of breast cancer and developed an assay for the ER in San Antonio. He readily agreed to measure ER in all of the Hershey patients on AG.

In the initial observational studies, the PSHG enrolled 147 breast cancer patients on AG/HC therapy and established its efficacy [14]. After that, to test its relative effectiveness, the PSHG set up two randomized, controlled trials, one comparing medical adrenalectomy to surgical adrenalectomy and the other to tamoxifen. One of the parameters to follow, was the healing of lytic lesions in bone. This required looking at several serial X-rays lined up on the view box. Our Endocrine Fellow Dr. Tom Worgul helped to organize all of this at Hershey. Dr.Samuel Wells at Duke spent about 72 h consecutively setting up all of the x-rays in order and Wells and an outside expert reviewed them blindly. Analysis of these and other parameters resulted in a New England Journal of Medicine article demonstrating that medical adrenalectomy was not inferior to surgical adrenalectomy [15]. This article led to the demise of surgical adrenalectomy for the treatment of breast cancer. The controlled trial of medical adrenalectomy versus tamoxifen also showed that the medical adrenalectomy was equally as effective as tamoxifen.

**Fig. 6:**
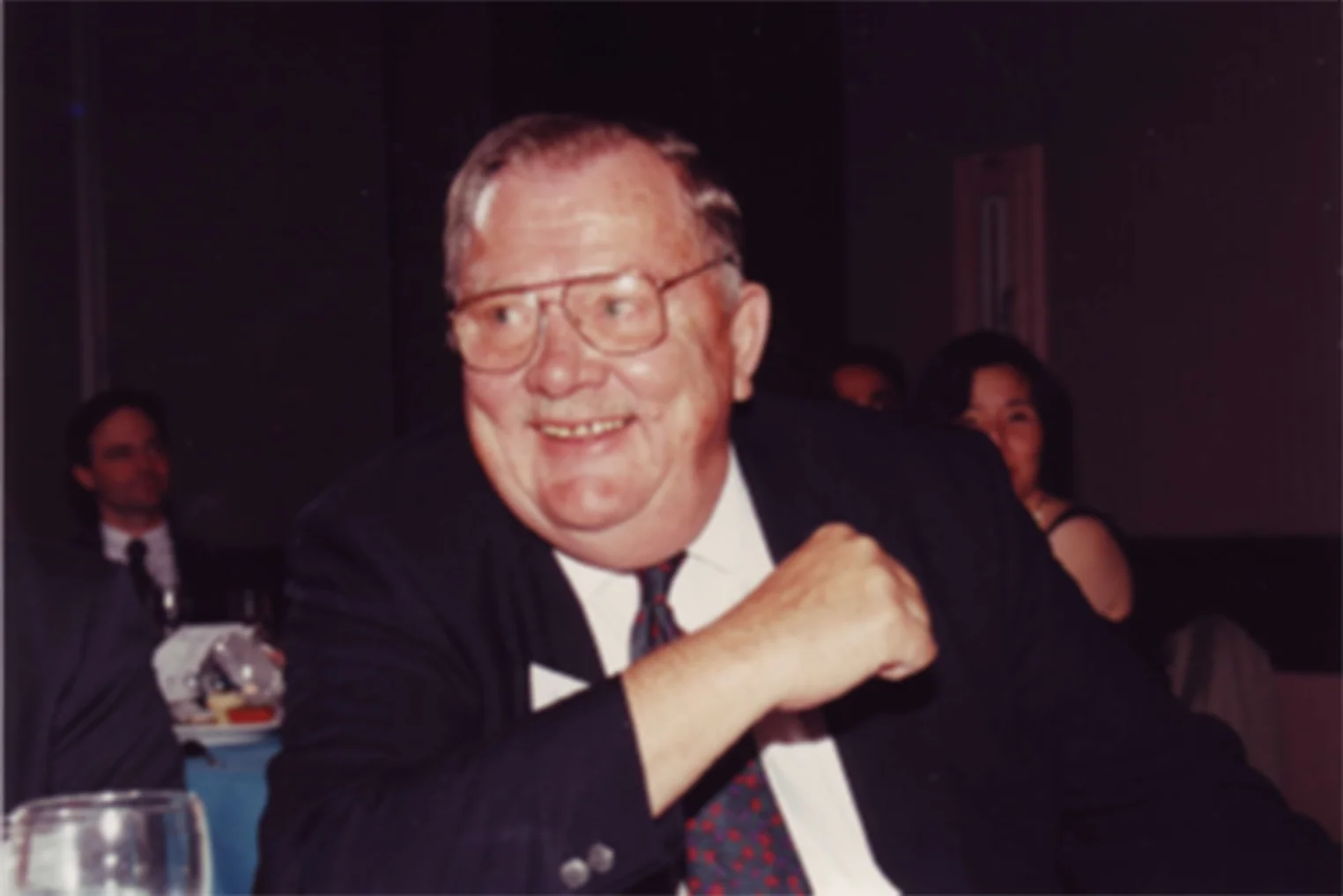
“Finn” Siiteri had more ideas than he could pursue himself but he encouraged many others to follow through on his ideas. He gave Dr Santen the idea that aminoglutethimide was an aromatase inhibitor

**Fig. 7:**
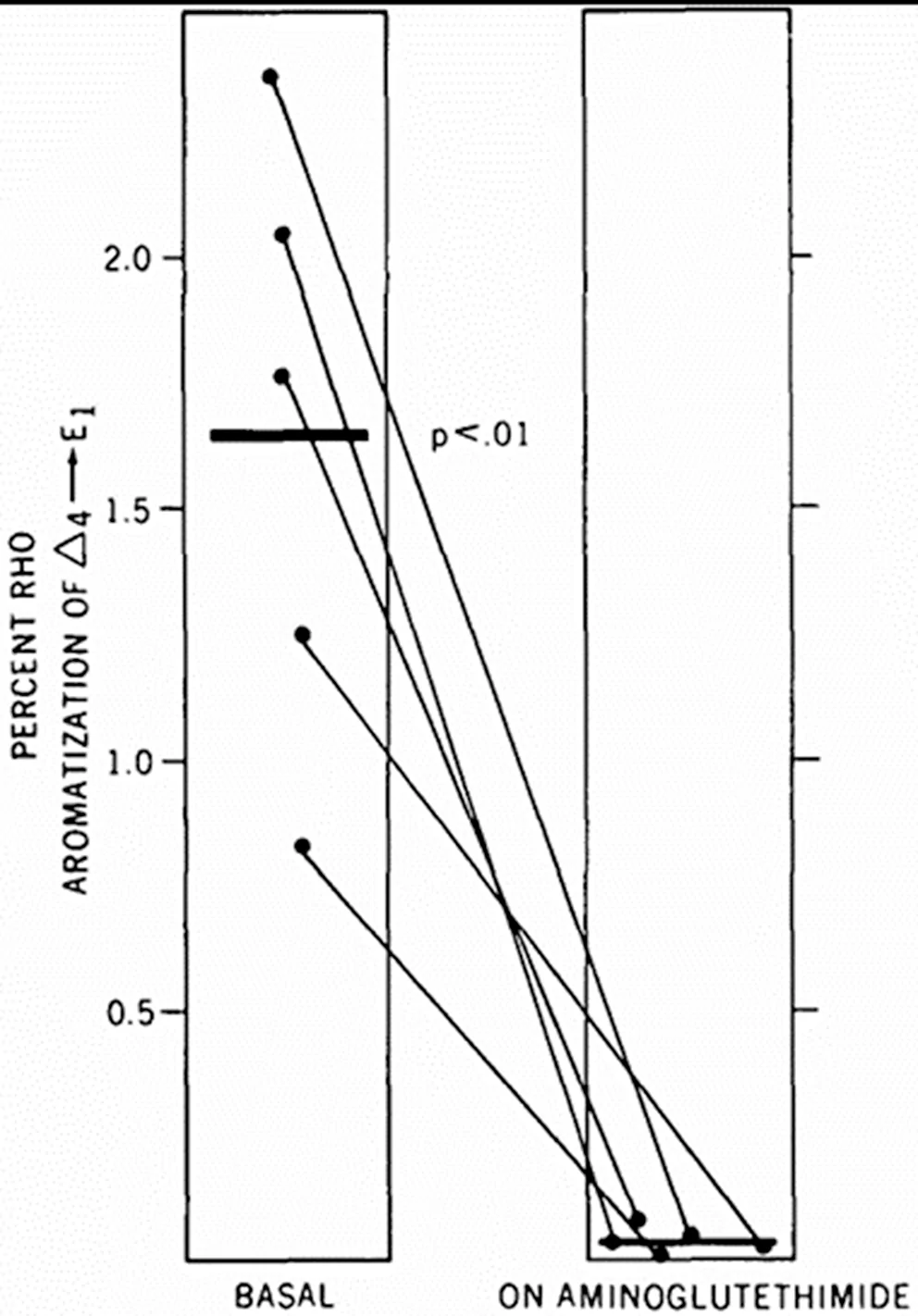
The figure shows the reduction of aromatase with aminoglutethimide. This was the first demonstration that an aromatase inhibitor could block aromatase, with inhibition of the conversion of androstenedione to estrone in postmenopausal women. The study involved the infusion of ^3^H androstenedione and ^14^C estrone into patients under equilibrium conditions and then purifying the products. This allowed for the demonstration that the Rho value (percent conversion of one compound into another under equilibrium conditions) before and after administration of aminoglutethimide was markedly inhibited. The steps necessary for steroid purification and the study of these patients took almost 5 years and many liters of urine collected [16]. Reprinted from JCEM [16] with the permission of the publisher

An interesting incident explains why we ultimately proved that AG mainly worked as an aromatase inhibitor. Dr. Penti (Finn) Siiteri came as a visiting professor to Hershey and gave a lecture. After that, he came to Santen’s office to meet with him. The first thing Siiteri said was “Dick you do not know what you are doing with aminoglutethimide”. He then said that he had published an abstract in 1967 that showed that AG was an aromatase inhibitor and that we should demonstrate in postmenopausal women that in fact this was the case. He told us how we should have 72 h of urine collected in patients given tritiated androstenedione and C14 estrone. It would then take about 22 steps to purify the steroids so that we could determine the percent conversion of androstenedione to estrone under equilibrium conditions. Dr. Steven Santner spent five years developing the necessary techniques. Santner then demonstrated that AG blocked the aromatase enzyme, the last step in estrogen synthesis, by 95 to 98% [16]. This clearly was the mechanism for blocking estrogen production in our patients and was also the reason why the drug was effective in treating breast cancer. We published this observation as the first demonstration of blockade of aromatase in postmenopausal women [16]. We abandoned the term **“Medical adrenalectomy”** and thereafter use the phrase **“Aromatase Inhibitor Therapy”** (Figs. 6, 7).

As the Ciba-Geigy pharmaceutical company had originally developed AG, the company became very interested in its development for breast cancer and invited Santen to Basel to present the PSHGP data. As described in detail in a later paragraph, the CIBA-Geigy leadership suggested holding an International Meeting to discuss the general concept of aromatase inhibitors. The meeting was held at Key Biscayne Florida with approximately 150 investigators in attendance. Dr. McGuire led much of the discussion. The proceedings were published in the journal **Cancer Research which** directed much attention to the concept of aromatase inhibition [8, 17–21].

It was clear at that time that AG was a non-selective aromatase inhibitor, blocking HC production as well as the estrogens. Notably, Dr Ronald Steele at Ciba-Geigy, had developed a more selective aromatase inhibitor which did not block cortisol production. This drug was called fadrozole, an imidazole-based compound, that was ultimately approved for treatment of breast cancer in Japan but never in the United States [22–24]. Demers’s laboratory demonstrated early on that fadrozole blocked aromatase but also caused a lowering of aldosterone as well through partial blockade of the 18-hydroxylase enzyme [25]. Demers and Lipton traveled to basal Switzerland, to share their findings with the Ciba-Geigy staff and particularly the lead investigator, Ajay Bhatnager, who was apparently unaware of this blockade causing mineralocorticoid deficiency. Ciba-Geigy stopped their clinical trials with fadrozole and eventually replaced this with another imidazole compound called letrozole which was a highly selective inhibitor of aromatase which did not have HC or aldosterone lowering effects.

**Fig. 8:**
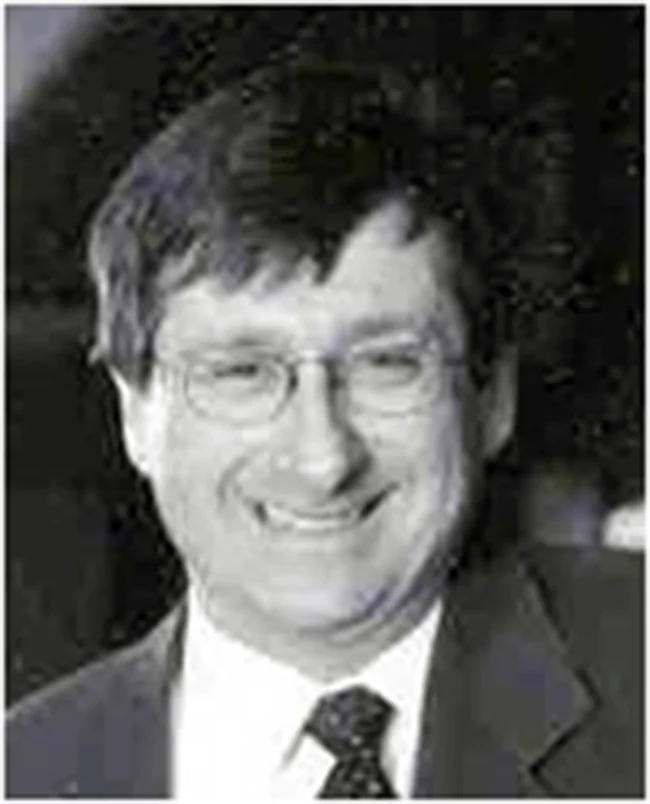
Dr. Paul Plourde; Endocrine Fellow at Penn State Hershey who went on to develop anastrozole for AstraZeneca and got approval from the FDA in 45 days, still a record for the time from application to approval

One of our fellows trained at Hershey, Paul Plourde, became employed by AstraZeneca and developed another aromatase blocking drug called anastrozole. The Demers laboratory performed some of the estrogen measurements for the Astra-Zeneca clinical trials and Santen and Demers consulted with Plourde to help Astra-Zeneca develop this medication. After submission to the FDA, anastrozole was approved after only 45 days. The rapid time to approval resulted because President Bill Clinton convinced the FDA at that time to initiate a fast-track method to approve cancer medications (Fig. 8).

As mentioned above, Ciba-Geigy had developed another aromatase inhibitor called letrozole [26, 27]. This compound was more potent than anastrozole but was not approved by the FDA for another year after the anastrozole approval. Both drugs were shown to be quite effective as aromatase inhibitors by the Demers CEL lab as well as by other investigators. A key question was to be addressed was whether aromatase inhibitors were superior to tamoxifen. Notably, there were many investigators that were initially skeptical about aromatase inhibitors and doubted that they might be more effective than tamoxifen. Two very large multi-center trials addressed this question and demonstrated that aromatase inhibitors were clinically more effective in treating breast cancer than tamoxifen [28, 29]. At that point, aromatase inhibitors became the standard of care for postmenopausal women with ER+ breast cancer.

Based on all of these studies, the PSHG became known as the initiating team for treating breast cancer patients with aromatase inhibitors. As a result, the Susan Komen Foundation presented the Brinker International Award to Santen in 1990, the first award given to anyone for development of aromatase inhibitors. It should be noted that other investigators, Dr Ralph Cash in Detroit, Michigan and Dr Ken Gale at the Upstate Medical Center in Syracuse, had worked on AG at the same time as the PSHG. However, Cash and Gale considered the AG regimen to be medical adrenalectomy and were not aware that AG was an aromatase inhibitor.

**Fig. 9:**
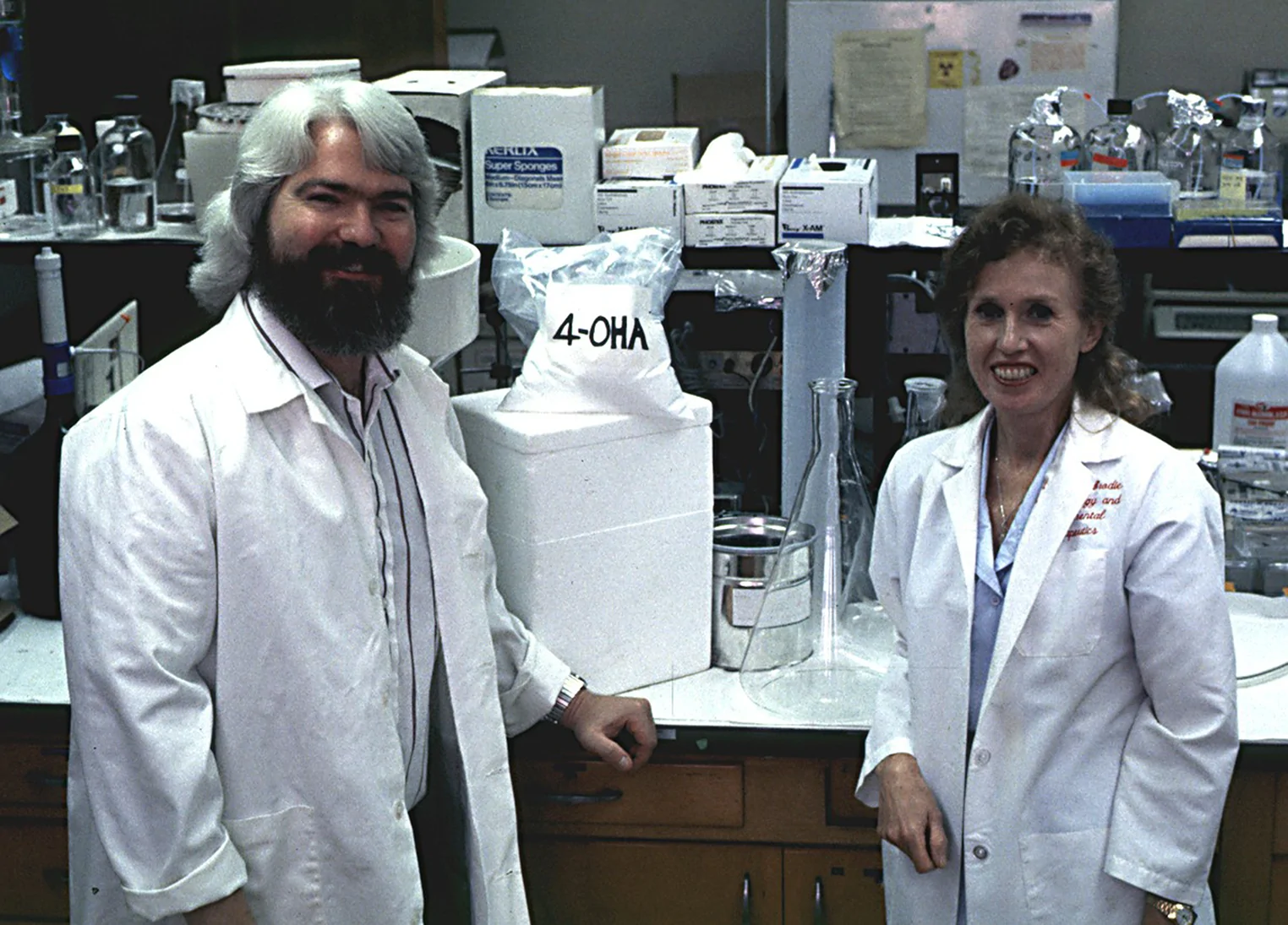
Dr Angela Brodie on the right developed the selective aromatase inhibitor 4-OH-androstenedone. She received the $500,000 General Motors Kettering award for this work The bag contains 4-OH A (4-hydroxy-androstenedione). The name of the laboratory technician on the left cannot be recalled at this point

Dr. Angela Brody and her husband Harry at the University of Maryland also had also been developing aromatase inhibitors since 1970 and identified in their research a steroid inhibitor of aromatase, 4-OH-androstenedione [30]. This was the first selective aromatase inhibitor, namely an agent which blocked only aromatase. This steroid compound was in development before the imidazole agents, anastrozole and letrozole. In contrast AG was non-selective and blocked several enzymatic steps. Along with Dr Paul Goss in the UK, Brodie conducted clinical trials with 4-OH-androstenedione (also called formestane) [31–34]. On this basis, Dr. Angela Brody won the General Motors Kettering prize of $500,000 in 2005 as the first individual to develop and test a selective aromatase inhibitor, 4-OH androstenedione (Fig. 9).

Several lessons were learned during development of the aromatase inhibitors. The first was that anyone who had ideas at a brand-new medical school such as Penn State Hershey would more easily be accepted by the new faculty. No one was there to say “we had tried that before and it did not work”. The reaction was “you have a new idea go ahead and see if it works” The second concept is that it takes a team and a strong collaborative effort to carry out comprehensive studies. Drs. Allen Lipton, Harold Harvey, Steven Santner, Larry Demers, and the endocrinology fellows all contributed to the aromatase inhibitor development. The steroid laboratory that Gene Samojlik developed was instrumental in allowing the demonstration that an adrenal inhibitor could block estrogen production. The collaboration of Dr. Samuel Wells at Duke provided an additional source of patients and clinical research acumen.

**Fig. 10:**
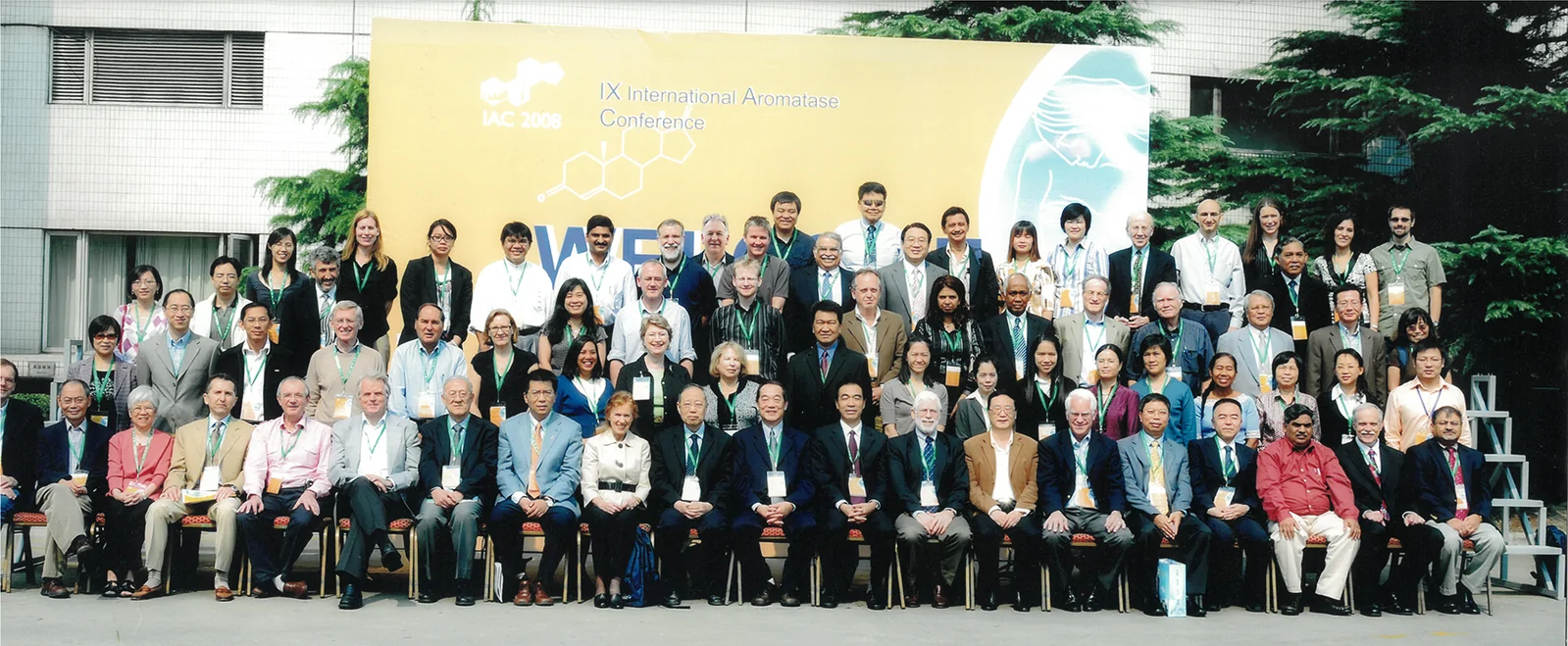
This is a photo of all of the attendees of International Aromatase meeting IX

Once our AG studies were mature, the Ciba-Geigy company wanted to disperse this information into the national and international oncology communities. They asked Santen, Lipton, and Harvey to arrange a meeting focusing on aromatase inhibitors for the treatment of breast cancer. As noted above, this was held in 1981 at Key Biscayne in Miami Florida. The majority of key opinion key leaders in the field of human breast cancer therapy and in the basic science of aromatase inhibitors were all invited. This initial aromatase meeting was felt to be quite helpful to investigators working in the field. Accordingly, 12 additional international aromatase meetings were held in various cities including Shanghai, Prague, Bologna, Houston, Miami, Houston, Edinburg, and multiple other cities (see appendix for full list). Each of these aromatase meetings was published in peer-reviewed medical literature, and all of this enhanced the development and acceptance of aromatase inhibitors. At the same time, these meetings allowed a group of investigators to get to know each other well, to collaborate on multiple aspects of the development of aromatase inhibitors, and to established many friendships. Shown below are attendees at Aromatase IX. The list of the entire 13 International Aromatase Meetings and the published proceedings is included at the end of this treatise (Fig. 10).

## Summary

The PSHG played a large role in the initial development of non-selective aromatase inhibitors for the treatment of breast cancer which led later to selective inhibitors. The basic and clinical studies conducted led to a greater understanding of the role of aromatase inhibitors and to the development of resistance to these agents. The PSHG and later studies by Santen’s basic science team and collaborators led to multiple peer reviewed publications [3–12, 14–27, 31, 35–64] The history is one of collaboration, interpersonal interactions, perseverance, and ability to focus on a goal. All of the participants in the PSHG had a real sense of adventure in working at a newly minted medical center. This manuscript is only a part of the total history of studies on aromatase as published in an extensive review.

## Supplementary Information

Below is the link to the electronic supplementary material.

Supplementary Material 1

## Data Availability

No datasets were generated or analysed during the current study.

## Abbreviations

PSHB: Penn State Hershey Group
ER: Estrogen receptor α
PgR: PgR progesterone receptor
AI: Aromatase inhibitor
AG: Aminoglutethimide
HC: Hydrocortisone
