# Supplementary material for: History of the early development of aromatase inhibitors for treatment of breast cancer
Source: Breast Cancer Res Treat. 2026 Aug 20;219(1):1. doi: 10.1007/s10549-026-08059-4 (PMC13493455; doi:10.1007/s10549-026-08059-4)
Supplement: Supplementary file 1 [file 10549_2026_8059_MOESM1_ESM.docx]

1^st^ International aromatase conference Key Biscayne, Florida December 1981 “International Symposium on aromatase: New Perspectives for Breast Cancer Cancer Research volume 42 Santen RJ, Lipton A, Harvey H eds. Cancer Research 42(8) supplement 3267s-3386s

2^nd^ International Aromatase meeting Miami Florida , March 4-7, 1987 published in Steroids volume 50 (1-3) “Proceedings of the Second International Conference on aromatase eds Siiteri PK and Gualtieri MD

3d International aromatase conference , Bologna , Italy June 14-17, 1992 “ Basic and Clinical Aspects of aromatase “ published in Journal of Steroid Biochemistry and Molecular biology 1993 44(4-6) 321-696, 1993 Brodie A, Brodie HB, Callard G, Robinson C and Santen R eds

4^th^ International aromatase conference Tahoe City , California July 7-11.1996 Journal of Steroid Biochemistry and Molecular Biology 61(3-6) 107-426, 1997

5^th^ International Aromatase Conference. Prague, Czech Republic. 3-6 September 1998. [Proceedings of the International Symposium on Aromatase and its Inhibition: New Biology and Clinical Perspectives.](https://erc.bioscientifica.com/view/journals/erc/6/2/10744463.xml)

published Endocrine-related Cancer 6, number 1, pages 127-333, 1999

6^th^ International Aromatase meeting, Port Douglas .Australia November 3-8, 2000 Published as “Aromatase 2000” Simpson ER and Pasqualini JR eds. Proceedings of the International Symposium on Aromatase and its Inhibition : new biology and clinical perspectives Journal of Steroid Biochemistry and Molecular Biology 79(1-5), pgs 1-328, 2001

7^th^ International Aromatase Conference Kyoto , Japan, Japan Oct 26-30, 2002 Proceedings edited by Dr Mitch Dowsett and Angela Brodie Published in Journal of Steroid Biochemistry and Molecular Biology 86(35) 205-555, 2002

8^th^ International Aromatase Conference, Sept 17, 200 Baltimore, Maryland organized by Dr Angela Brodie and published in the Journal of Steroid Biochemistry and Molecular Biology 106(1-5) pg 1-18 Brodie AMH and Pasqualini JR eds. “Aromatase 2026”

9^th^ International Aromatase meeting , Shanghai, China October 13-17, 2008, articles from this conference are published as individual articles and not in a complete volume of proceedings

10^th^ International Aromatase meeting , Edinburgh , Scotland Sept 6-8, 2010 Published in the Journal Steroids “10^th^ International Aromatase Conference Proceedings,2011, Dowsett M, P Lonning P editors 76(8) 729

11^th^ International Aromatase Conference, Houston, TX, June 27-28, 2012, Chair: Rajeshwar Rao Tekmal and co-chair: Aman Buzdar, held at MD Anderson Cancer Center ndividual articles published

12^th^ International Aromatase Conference, Chicago, IL, June 25-26, 2014; Chair: Rajeshwar Rao Tekmal, Co-chair: Sardar Bulan, Held at Northwestern University individual articles published

13^th^ International Aromatase Conference, Held at Univ of Texas Health at San Antonio, TX, December 10-11, 2016. Chair: Rajeshwar Rao Tekmal in conjunction with the San Antonio Breast Cancer Symposium. Individual articles published separately
